# Persistence and adherence to sodium-glucose co-transporter 2 inhibitor monotherapy among patients with type 2 diabetes mellitus: a retrospective study based on a Japanese claims database

**DOI:** 10.1007/s13340-025-00821-1

**Published:** 2025-05-29

**Authors:** Junichi Mukai, Manabu Akazawa, Yuji Yoshiyama, Rie Kubota

**Affiliations:** 1 Division of Clinical Pharmacy (Laboratory of Clinical Pharmacy Education) and Research and Education Centre for Clinical Pharmacy, School of Pharmacy, Kitasato University, 5-9-1 Shirokane, Minato-ku, Tokyo, 108-8641 Japan; 2 Department of Public Health and Epidemiology, Meiji Pharmaceutical University, 2-522-1 Noshio, Kiyose, Tokyo 204-8588 Japan; 3 Division of Clinical Pharmacy (Laboratory of Pharmaceutical Health Care and Sciences in Community Pharmacy Practice) and Research and Education Centre for Clinical Pharmacy, School of Pharmacy, Kitasato University, 5-9-1 Shirokane, Minato-ku, Tokyo, 108-8641 Japan

**Keywords:** Sodium-glucose co-transporter 2 inhibitor, Adherence, Persistence, Type 2 diabetes, Observational study, Retrospective study

## Abstract

The aims of this retrospective study were to examine persistence/adherence rates to sodium-glucose co-transporter 2 inhibitors (SGLT2i) monotherapy in patients with type 2 diabetes (T2DM) and identify factor(s) affecting persistence/adherence. Claims data on patients with T2DM newly using SGLT2i monotherapy from the JMDC database between October 2017 and September 2020 were analyzed. Persistence without a 90-day gap was calculated from the index date until the time of discontinuation of SGLT2i in a 1-year follow-up. Adherence was calculated using the proportion of days covered (PDC). Baseline characteristics were examined as potential factors affecting persistence/adherence using a multivariate logistic method. The present study identified 2172 new users of SGLT2i monotherapy. The persistence rate to SGLT2i after 365 days was 61.0%. Mean PDC was 71.2%, and 58.3% of patients adhered to treatment. A multivariate logistic regression analysis showed that an older age, hypertension, dyslipidemia, and hyperuricemia were associated with a lower risk of the discontinuation of SGLT2i monotherapy, while an older age, hypertension, dyslipidemia, and hyperuricemia were associated with a lower risk of poor adherence. The present study identified several factors that reduced the risk of discontinuation/poor adherence to SGLT2i monotherapy in patients with T2DM. An older age, hypertension, dyslipidemia, and hyperuricemia were common factors for a lower risk of discontinuation/poor adherence.

## Introduction

Patients with type 2 diabetes mellitus (T2DM) need to adhere to and persist with treatment with oral antidiabetic drugs (OAD) because poor adherence may lead to poor clinical outcomes, such as increased mortality or hospitalization [1, 2]. Previous studies reported on adherence/persistence to OAD in Japanese patients with T2DM. A Japanese hospital database analysis to evaluate persistence with OAD in patients with T2DM by Kadowaki et al. revealed that among six OAD other than sodium-glucose co-transporter 2 inhibitors (SGLT2i), persistence to Dipeptidyl peptidase-4 inhibitors (DPP4i) was the longest with no gap for > 30 days [3]. A questionnaire survey by Hayashi et al. on medication adherence to OAD indicated good adherence by Japanese patients with T2DM to DPP4i and biguanide monotherapy with any dosing regimen [4]. Additionally, a cohort study by Kurtyka et al. using a Japanese claims database that included new DPP4i users showed that adherence to DPP4i monotherapy in patients with T2DM was better in Japan than in the United States [5]. In a systematic review that summarized 22 observational studies on SGLT2i, Ofori-Asenso et al. concluded that adherence and persistence to SGLT2i were poor; however, the majority of studies (12 out of 22) in this review were performed in the United States, and Japan was not included [6]. One Japanese study that examined persistence/adherence to SGLT2i in untreated patients with T2DM using two claims databases showed that the 12-month persistence rate of SGLT2i monotherapy was lower than that of DPP4i monotherapy (53.5% vs 67.4%) [7]. However, the sample size was small and the study may have been performed too early to reflect current SGLT2i prescriptions because it included data within one year of the launch of SGLT2i; a newly approved drug is generally limited to 14 days of supply due to safety concerns for the first year in Japan. The Japan Diabetes Society recommends the initiation of a single low OAD dose for patients with T2DM [8]. Previous findings on OAD using a healthcare claims database showed that a younger age, the presence of comorbidities, and no concomitant drugs were factors worsening persistence/adherence [5, 9, 10]. However, to the best of our knowledge, few studies in Japan have examined factor(s) affecting persistence/adherence to SGLT2i monotherapy. To obtain a more detailed understanding of treatment with SGLT2i, a retrospective study using a Japanese claims database was performed to examine persistence and adherence rates to SGLT2i monotherapy in patients with T2DM and also to identify factor(s) affecting persistence/adherence.

## Methods

### Data source

The present study was conducted using the JMDC database (https://www.jmdc.co.jp/en/jmdc-claims-database/), which stores healthcare claims data from approximately 14 million beneficiaries as of February 2022. The JMDC database only covers employees of medium/large businesses and their dependents with employee health insurance; however, it has high traceability due to the storage of healthcare claims. These data have anonymized information on the characteristics of individuals < 75 years (those > 75 years switch from health insurance to the medical care system for the late elderly in Japan), including their diagnoses, prescriptions, and the medical institutions at which they were treated. Data collected between October 2017 and September 2020, which included 68,085 patients diagnosed with T2DM (international classification of disease (ICD)-10 code: E11) and prescribed SGLT2i (anatomical therapeutic chemical (ATC) code: A10P), were used.

### Study design and population

This was a retrospective study with a new user design, which excluded prevalent antidiabetic agent users [11]. The index date was defined as the date of the first prescription for SGLT2i monotherapy. Patients were included in the cohort if they had both 6-month (180-day) pre-index and 1-year (365-day) post-index periods. New users, defined as patients newly prescribed SGLT2i without previously being prescribed antidiabetic agents (ATC code: A10) for the pre-index period, were included in the cohort. Our cohort assumed that new users of SGLT2i were newly diagnosed with T2DM. Patients were excluded if they (1) had no prescription date for antidiabetic agents; (2) started any combination therapy with an SGLT2i and another antidiabetic agent; (3) started monotherapy with a fixed-dose combination tablet of SGLT2i at the index date; (4) only had one prescription for SGLT2i for the post-index period [12]; (5) or had an outpatient visit < 2 times within the pre-index period. The follow-up period was the index date plus 365 days.

### Definitions of outcomes

Persistence was calculated for each individual from the index date until the time SGLT2i monotherapy was discontinued. The initiation of combination therapy with SGLT2i was regarded as non-persistence. A persistence study must include a permissible gap as the maximum allowable period without treatment [13]. The event of SGLT2i discontinuation was a 90-day gap between two prescription dates; a permissible 90-day gap was frequently used in a meta-analysis, which included observational studies that evaluated persistence to SGLT2i [6]. When discontinuation occurred, the date when SGLT2i was discontinued was calculated to include the days of supply of SGLT2i to the last prescription date. If SGLT2i was continued without a 90-day gap between two prescription dates, censoring was at the end of a one-year (365-day) follow-up. Patients censored after 365 days were defined as persistent; the remaining were defined as non-persistent. We assessed the first discontinuation only. The restart rate of SGLT2i was calculated based on the number of patients who had discontinued SGLT2i but subsequently received at least one prescription of SGLT2i within the post-index period; restarters were not counted in the cohort. Adherence was calculated using the proportion of days covered (PDC), which was defined as the total number of SGLT2i-covered days divided by 365. The total number of SGLT2i-covered days was the total days of the supply of SGLT2i without doubling the count if the prescription was refilled before the supply ran out. To calculate PDC, the discontinuation date for SGLT2i was defined as the date achieved by adding days of the supply of SGLT2i to the last prescription or at the end of the 1-year (365-day) follow-up, whichever occurred first. In this case, the 90-day treatment gap was not considered. Patients with PDC ≥ 80% were defined as adherent and with PDC < 80% were defined as poor adherent because this threshold is regarded as a reasonable cut-off point for good adherence [14, 15], and the adherence rate for 12 months was also calculated as a percentage.

All residents of Japan must enroll in the national health insurance system and generally pay 30% of their medical fees, with the remaining 70% being covered by the insurance system. A medical institution (i.e. clinic or hospital) may be selected without referral from a general practitioner. The standard number of days for which a prescription is supplied ranges between 1 and 90 days in Japan.

### Covariates

The following factors were considered to affect persistence and adherence to SGLT2i therapy: the calendar year at the index date, age at the index date, sex, the number of outpatient visits, the mean interval between outpatient visits, the number of concomitant drugs, hypertension, dyslipidemia, depression, obesity, hyperuricemia, diabetic nephropathy, and the Charlson comorbidity index (CCI). The number of concomitant drugs that were counted included the following: renin-angiotensin system agents (the ATC code for C09), beta blockers (the ATC code for C07), calcium channel blockers (the ATC code for C08), diuretics (the ATC code for C03), and lipid-lowering agents (the ATC code for C10). The CCI is a disease risk score that estimates the risk of death from comorbid conditions (e.g. the presence or absence of diabetic complications); the greater the sum of the index score weighted based on disease severity, the greater the disease burden on each patient [16]. These factors, except for calendar year, age, and sex, were measured for six months before the index date. Previous studies reported relationships between the following factors and poor adherence: sex [10], age and the number of concomitant medications [5, 10], the number of outpatient visits [17], chronic conditions [18], and depression and dementia [19]. Dementia is regarded as one of the elements of the CCI. Each claim represented one outpatient visit. Hypertension and dyslipidemia were identified as the ICD-10 codes for E78 and I10, respectively. Similarly, depression was identified as the ICD-10 codes for F32 and F33. Furthermore, obesity and hyperuricemia were labeled as the ICD-10 codes for E66 and E790 and diabetic nephropathy as the codes for E112, E142, N083, and N189. The CCI was also calculated using the ICD-10 codes. Flags for suspected diseases, such as a tentative diagnosis, were excluded to avoid misclassification.

### Statistical analysis

Descriptive data were expressed as means and standard deviations (SD) for continuous variables and as frequencies and percentages for categorical variables. If the JMDC database stored a date as six digits (e.g. a patient’s birthday, the start and stop dates of the patient’s observation period, and the dates of outpatient visits), an arbitrarily chosen two-digit number, 15, representing the date was entered after the six-digit number representing the year and month used in the JMDC database. For example, 20,220,815 represents August 15, 2022. The Kaplan–Meier method was used to assess 365-day persistence to SGLT2i monotherapy. If persistence to the index treatment extended beyond the end of the follow-up period, patients were censored. Regarding binary outcomes for poor persistence and adherence, a multivariate logistic regression model was used to assess odds ratios for each independent variable affecting persistence or adherence to SGLT2i monotherapy. We examined the multicollinearity assumption for the factors included. All factors were included in both analyses because of the lack of multicollinearity. A *p* value < 0.05 was considered to be significant. All statistical analyses were performed with Stata/MP 17.0 version (Stata Corp, College Station, TX, USA).

### Additional analyses

We performed a sensitivity analysis to assess persistence and restart rates in terms of a 30-day gap: a 30-day gap was defined as the median days of supply of SGLT2i included in the post-index period of new users starting SGLT2i monotherapy. We performed linear regression analysis to evaluate the relationship between age or interval between outpatient visits and medication persistence/adherence.

### Ethics

The Kitasato Institute Hospital Research Ethics Committee waived ethical approval for this study because it was not applicable for Ethical Guidelines for Medical and Biological Research Involving Human Subjects (5th July 2022). Informed consent was not needed because of retrospective nature of the study.

## Results

**Fig. 1:**
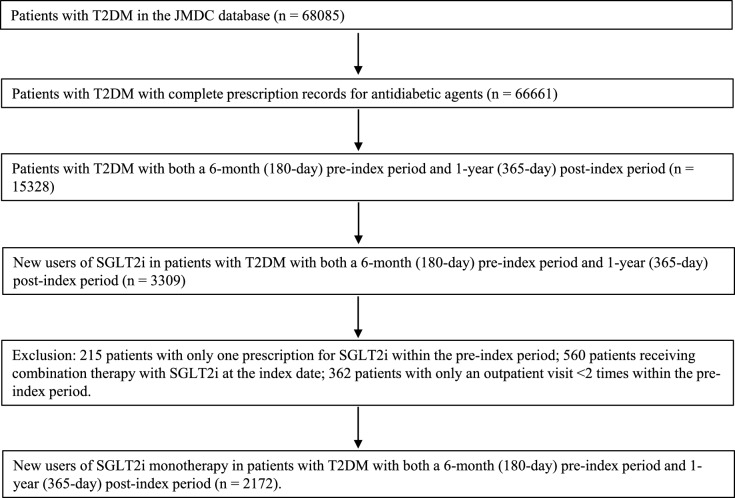
Flow chart for the selection of new users of SGLT2i monotherapy (n = 2172). *SGLT2i* sodium-glucose co-transporter 2 inhibitor, *T2DM* type 2 diabetes mellitus

Among 68,085 patients with T2DM, 3309 were new users of SGLT2i therapy, identified at the index date. A total of 215 patients were subsequently excluded because they only had one prescription of SGLT2i in the post-index period and the remaining 560 new users received combination therapy with SGLT2i at the index date. Furthermore, of 2534 patients, 362 with an outpatient visit < 2 times within the pre-index period were excluded. Therefore, 2172 new users of SGLT2i monotherapy at the index date were identified (Fig. 1).

### Baseline characteristics

Table 1 shows patient characteristics at baseline. The mean age of patients was 51.9 years and 73.4% were male. During the 180-day pre-index period, the mean number of outpatient visits was 6.2 and renin-angiotensin system agents and lipid-lowering agents were prescribed to 40–50% of patients. More than 60% of patients had a comorbidity of hypertension or dyslipidemia and the mean CCI score was 1.2.

**Table 1:** Baseline characteristics (n = 2172)

Variables	
Calendar year at the index date, n (%)	
2018	995 (45.8)
2019	1177 (54.2)
Sex, male, n (%)	1595 (73.4)
Age at the index date, years, mean (SD) (range)	51.9 (9.1) (20.5–73.9)
Number of outpatient visits, preceding 180 days, mean (SD)	6.2 (4.4)
Mean interval between outpatient visits, preceding 180 days, days, mean (SD)	33.7 (24.4)
Concomitant drugs preceding 180 days: the ATC code, n (%)	
^a^Renin-angiotensin system agents: C09	973 (44.8)
Beta blockers: C07	280 (12.9)
Calcium channel blockers: C08	649 (29.9)
Diuretics: C03	204 (9.4)
^a^Lipid-lowering agents: C10	1048 (48.3)
^b^Number of concomitant drugs, preceding 180 days, mean (SD)	1.5 (1.2)
Comorbidity preceding 180 days: the ICD-10 code, n (%)	
Hypertension: E78	1327 (61.1)
Dyslipidemia: I10	1413 (65.1)
Depression: F32-33	185 (8.5)
Obesity: E66	124 (5.7)
Hyperuricemia: E790	464 (21.4)
Diabetic nephropathy: E112, E142, N083, N189	170 (7.8)
^c^Charlson comorbidity index, preceding 180 days, mean (SD) (range)	1.2 (1.4) (0–13)

*SD* standard deviation, *ATC* anatomical therapeutic chemical, *ICD* international classification of disease

^a^Included compound drugs

^b^Included renin-angiotensin system agents, beta blockers, calcium channel blockers, diuretics, and lipid-lowering agents

^c^The value was low because new users were defined as patients without any other antidiabetic drugs prescribed in the preceding 180 days

### Persistence

**Fig. 2:**
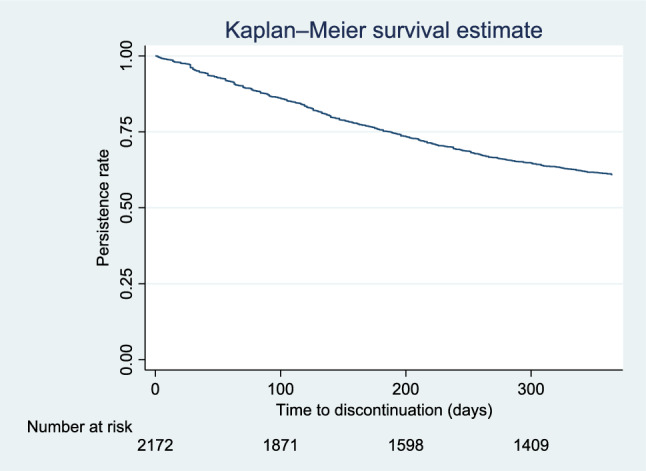
Kaplan–Meier estimate of the time to the discontinuation of SGLT2i monotherapy (n = 2172). *SGLT2i* sodium-glucose co-transporter 2 inhibitor

The mean 1-year follow-up period for SGLT2i monotherapy was 281.7 days; patients with follow-up periods longer than 1 year were censored. We identified 1588 persistent patients and 584 non-persistent patients. The 1-year incidence for non-persistence was 505.8 per 1000 person-year. No patients were missing data for the follow-up periods. The persistence rate to SGLT2i monotherapy at 365 days was 61.0% (Fig. 2). The restart rate of SGLT2i after discontinuation within the post-index period in terms of a 90-day gap was 45.4% (385 patients). A multivariate logistic regression analysis after adjustments for all factors showed that an increase in age (shown at one-year intervals), hypertension, dyslipidemia, and hyperuricemia were associated with a lower risk of the discontinuation of SGLT2i monotherapy (Table 2).

**Table 2:** Factors affecting discontinuation

Variables	Persistent n = 1588	Non-persistent n = 584	Crude odds ratios (95%CI)	^b^Adjusted odds ratios (95%CI)
	n (%)	n (%)		
Calendar year 2018 at the index date	723 (72.7)	272 (27.3)	0.996 (0.838–1.185)	0.966 (0.808–1.153)
Sex, male	1193 (74.8)	402 (25.2)	0.816 (0.672–0.990)	0.858 (0.698–1.055)
^a^Age at the index date, years	52.6 (8.6)	50.0 (9.9)	0.966 (0.956–0.975)	0.968 (0.958–0.978)
^a^Number of outpatient visits, preceding 180 days	6.2 (3.6)	6.3 (6.1)	1.005 (0.986–1.025)	1.007 (0.981–1.033)
^a^Mean interval between outpatient visits, preceding 180 days, days	33.4 (23.0)	34.7 (27.8)	0.999 (0.996–1.003)	0.999 (0.995–1.003)
^a^Number of concomitant drugs, preceding 180 days	1.5 (1.2)	1.2 (1.2)	0.868 (0.807–0.933)	1.069 (0.964–1.185)
Comorbidity, preceding 180 days				
Hypertension	1029 (77.5)	298 (22.5)	0.645 (0.541–0.770)	0.720 (0.568–0.913)
Dyslipidemia	1071 (75.8)	342 (24.2)	0.669 (0.559–0.801)	0.736 (0.602–0.901)
Depression	122 (66.0)	63 (34.1)	1.397 (1.032–1.890)	1.248 (0.893–1.745)
Obesity	84 (67.7)	40 (32.3)	1.021 (0.705–1.480)	1.026 (0.699–1.506)
Hyperuricemia	370 (79.7)	94 (20.3)	0.683 (0.549–0.849)	0.754 (0.597–0.951)
Diabetic nephropathy	131 (77.1)	39 (22.9)	0.864 (0.624–1.197)	0.935 (0.663–1.320)
^a^Charlson comorbidity index score, preceding 180 days	1.2 (1.4)	1.1 (1.4)	0.943 (0.885–1.004)	0.994 (0.927–1.065)

*SD* standard deviation, *CI* confidence interval

^a^Values are shown as mean (SD)

^b^The multivariate logistic regression model was adjusted for all variables in Table 2

### Adherence

The mean PDC was 71.2%, and 58.3% of patients adhered to treatment. We identified 1267 adherent patients (PDC ≥ 80%) and 905 poor adherent patients (PDC < 80%). A multivariate logistic analysis after adjustments for all factors showed that an older age (shown at one-year intervals), hypertension, dyslipidemia, and hyperuricemia were associated with a lower risk of poor adherence, while no factors were associated with a higher risk (Table 3).

**Table 3:** Factors affecting poor adherence

Variables	Adherent (PDC ≥ 80%) n = 1267	Poor adherent (PDC < 80%) n = 905	Crude odds ratios (95%CI)	^b^Adjusted odds ratios (95%CI)
	n (%)	n (%)		
Calendar year 2018 at the index date	581 (58.4)	414 (41.6)	0.996 (0.839–1.181)	0.972 (0.815–1.158)
Sex, male	934 (58.6)	661 (41.4)	0.966 (0.796–1.171)	0.988 (0.804–1.214)
^a^Age at the index date, years	53.1 (8.6)	50.3 (9.5)	0.966 (0.957–0.976)	0.971 (0.961–0.981)
^a^Number of outpatient visits, preceding 180 days	6.3 (3.6)	6.1 (5.4)	0.988 (0.968–1.009)	0.996 (0.973–1.021)
^a^Mean interval between outpatient visits, preceding 180 days, days	33.4 (23.0)	34.7 (27.8)	1.001 (0.998–1.005)	1.000 (0.996–1.004)
^a^Number of concomitant drugs, preceding 180 days	1.5 (1.2)	1.3 (1.2)	0.855 (0.796–0.919)	1.056 (0.954–1.169)
Comorbidity, preceding 180 days				
Hypertension	832 (62.7)	495 (37.3)	0.631 (0.530–0.752)	0.717 (0.568–0.907)
Dyslipidemia	877 (62.1)	536 (37.9)	0.646 (0.540–0.772)	0.742 (0.608–0.906)
Depression	97 (52.4)	88 (47.6)	1.299 (0.960–1.757)	1.285 (0.920–1.793)
Obesity	75 (60.5)	49 (39.5)	0.910 (0.628–1.318)	0.967 (0.659–1.417)
Hyperuricemia	299 (64.4)	165 (35.6)	0.722 (0.583–0.893)	0.792 (0.631–0.995)
Diabetic nephropathy	101 (59.4)	69 (40.6)	0.953 (0.693–1.311)	1.093 (0.780–1.532)
^a^Charlson comorbidity index score, preceding 180 days	1.3 (1.4)	1.1 (1.4)	0.898 (0.842–0.958)	0.950 (0.886–1.018)

*PDC* the proportion of days covered, *SD* standard deviation, *CI* confidence interval

^a^Values are shown as mean (SD)

^b^The multivariate logistic regression model was adjusted for all variables in Table 2

### Additional analyses

In the 30-day gap period, the mean 1-year follow-up of SGLT2i monotherapy was 264.5 days and the 1-year persistence rate was 53.8%. The restart rate of SGLT2i after its discontinuation within the post-index period for a 30-day gap was 57.5% (577 patients). Age was positively associated with good medication persistence/adherence such as PDC and the 1-year follow-up period for SGLT2i monotherapy (95% CI 3.71–6.10, *p* < 0.001, 95% CI 0.01–0.02, *p* < 0.001). The mean interval between outpatient visits was not associated with PDC or the 1-year follow-up period for SGLT2i monotherapy.

## Discussion

Using the JMDC database, a retrospective study on patients with T2DM was conducted to examine persistence and adherence rates to SGLT2i monotherapy and identify factor(s) affecting persistence/adherence. Persistence rates to SGLT2i without exceeding 90- and 30-day gaps at 365 days were 60.0 and 53.8%, respectively. Mean PDC at 365 days was 71.2%, and 58.3% of patients adhered to treatment.

Persistence and adherence rates in the present study were equivalent to those in a previous review [6]. An older age (shown at one-year intervals), hypertension, dyslipidemia, and hyperuricemia were factors increasing persistence and adherence to SGLT2i monotherapy.

A systematic review of 22 observational studies from eight countries (excluding Japan) revealed that the pooled persistence rate without exceeding a 90-day gap to SGLT2i therapies at 12 months was 58.9%, the pooled mean PDC at 12 months was 72.0%, and the pooled percentage of adherence at 12 months was 49.0% [6]. The present results showed that the persistence rate to SGLT2i without exceeding a 90-day gap at 12 months was 60.0%, the mean PDC at 12 months was 71.2%, and 58.3% of patients adhered to treatment. The results obtained on persistence and adherence rates in the present study, which was performed in Japan, were equivalent to those in a systematic review [6]; a study by McGovern et al., which was included in the review, reported the highest persistence rate of 69.5% at 1 year and the mean age of patients was 66.1 years [20]. Additionally, the present results were slightly worse than the findings reported by Kurtyka et al., which had the same gap period of 90 days in the JMDC database; the persistence rate to DPP4i monotherapy at 12 months was 72.2%, while the mean PDC and percentage of adherence were 76.6 and 67.2%, respectively [5]. These results support persistence and adherence rates to SGLT2i monotherapy at 12 months being slightly lower than those to DPP4i monotherapy [7]. Persistence depends on the gap periods of each study. Consistent with previous findings [21], the present results showed that the persistence rate to SGLT2i monotherapy with a 90-day gap period was higher than that with a 30-day gap period. The results obtained herein showed that even if a 30-day gap period was permissible, the persistence rate to SGLT2i monotherapy in patients with T2DM was higher than 50%.

The present study demonstrated that an older age reduced the risk of poor persistence and adherence, which is consistent with the findings of observational studies on patients with T2DM who received OAD [5, 10, 22]. Patients with T2DM who adhere to treatment are more likely to have good glycemic control [23], while those who do not are expected to have poor clinical outcomes [1, 2]. Based on these findings, older patients with T2DM who adhere to treatment may have relatively good glycemic control: however, our database was unable to capture hemoglobin A1c.

Comorbid conditions, such as hypertension, dyslipidemia, and hyperuricemia, were associated with a lower risk of the discontinuation of SGLT2i monotherapy: the adjusted odds ratio for hypertension was 0.72 (95% CI 0.57–0.91), that for dyslipidemia was 0.74 (95% CI 0.60–0.90), and that for hyperuricemia was 0.75 (95% CI 0.60–0.95). “The healthy adherer effect”, which indicates that patients with a chronic condition(s) are more likely to adhere to their therapies [24], appears to have contributed to these results because the majority of patients in the present study had chronic conditions, and older Japanese patients with T2DM are expected to have diabetic complications [25]. Additionally, one systematic review to summarize factors associated with good adherence to urate lowering therapy showed that adherent patients with gout had a high number of comorbidities, diabetes, and hypertension [26]. These findings partially support the present results. A previous study showed that hypertensive patients with controlled blood pressure were more likely to be adherent than those with uncontrolled blood pressure [27]. Furthermore, self-monitoring blood pressure is one of the interventions that achieves good adherence to anti-hypertensive medication [28]. Patients with hypertension or dyslipidemia may visually monitor their blood pressure or blood lipid levels. Neither blood pressure nor blood lipid levels at baseline were available for analysis, which may have been because our cohort included stable patients with hypertension or dyslipidemia. The reason why the odds ratios for hypertension, dyslipidemia, and hyperuricemia were lower than that for an older age currently remains unclear: the adjusted odds ratio for an older age was 0.97 (95% CI 0.96–0.98).

The present study showed that an older age, hypertension, dyslipidemia, and hyperuricemia were associated with persistence and adherence to SGLT2i monotherapy, which was consistent with that of other OAD. Kurtyka et al. and Miwa et al. reported that a younger age and comorbidity were associated with lower adherence and higher discontinuation to DPP4i monotherapy or DPP4i regimens [5, 9]. Tunceli et al. demonstrated that a younger age and no concomitant medications increased the likelihood of non-adherence to antihyperglycemic agent monotherapy, which included more than 70% metformin [10]. Therefore, these factors may be common with adherence and persistence to OAD.

Several interventions have been reported to improve adherence in patients with diabetes, including diabetes education, increasing counselling, and keeping regimens as simple as possible [29]. Although the present study showed that the number of outpatient visits was not associated with a higher adherence rate to SGLT2i monotherapy, previous Japanese studies, such as a cross-sectional study conducted by Okuno et al., demonstrated that patients with more frequent outpatient visits had a better relationship with their doctors and higher adherence rates [30]. Regarding simple dosing, a recent study showed that the 1-year persistence rate was higher with DPP4i than with α-glucosidase inhibitors [7]. Patients with chronic diseases who receive their first prescription are more likely to discontinue treatment at an earlier stage [31]. Negative perceptions of long-term medication, such as adverse event(s), may affect the adherence of a new user to chronic medication despite the benefits of treatment [32].

The strengths of the present study were the use of a large healthcare claims database and the majority of data being obtained from clinics. Japanese patients with T2DM generally visit a clinic [33]. Therefore, the results obtained herein may be generalized to patients with T2DM who visit clinics. However, there are several limitations that need to be addressed. The present results may not be generalized to all patients with T2DM because there may have been a selection bias due to the JMDC database including a small population of individuals aged 65 years or older and not those aged 75 years. This bias may have also distorted the true relationship between an older age and good adherence/persistence. Furthermore, our cohort included approximately 70% male patients. Another limitation is that although our study only targeted SGLT2i monotherapy, combination therapy with OAD is common in Japan. Unmeasured factors, such as the duration of diabetes, hemoglobin A1c, and the body mass index, may have affected the results obtained based on previous findings showing that hemoglobin A1c was associated with poor adherence [34]. Furthermore, reasons for the discontinuation of SGLT2i monotherapy were not examined in the present study. These factors and reasons for discontinuation were not identified due to the lack of relevant data in the JMDC database. Previous studies showed that the Morisky score was associated with medication adherence in patients with T2DM [4, 35]; however, we were unable to incorporate this variable due to the nature of the healthcare claims database. Since the present cohort had strict inclusion criteria assuming new users of SGLT2i, the relationship between previous experience with antidiabetic agents and adherence to SGLT2i was not investigated. Moreover, it is unclear whether patients actually took SGLT2i because the present study relied on prescription data.

## Conclusion

The present study identified several factors in patients with T2DM that contribute to good persistence and adherence to SGLT2i monotherapy. An older age, hypertension, dyslipidemia, and hyperuricemia were common factors for good persistence/adherence to this treatment.

## Data Availability

The datasets used and/or analyzed during the present study are available from the corresponding author upon reasonable request.

## Abbreviations

SGLT2i: Sodium-glucose co-transporter 2 inhibitor
OAD: Oral antidiabetic drug
DPP4i: Dipeptidyl peptidase-4 inhibitor
T2DM: Type 2 diabetes mellitus
CCI: The Charlson comorbidity index
SD: Standard deviations
CI: Confidence interval
PDC: The proportion of days covered
ATC: Anatomical therapeutic chemical
ICD: International classification of disease
